# Macauba (*Acrocomia aculeata*) Pulp Oil Prevents Adipogenesis, Inflammation and Oxidative Stress in Mice Fed a High-Fat Diet

**DOI:** 10.3390/nu15051252

**Published:** 2023-03-02

**Authors:** Cíntia Tomaz Sant’ Ana, Thaísa Agrizzi Verediano, Mariana Grancieri, Renata Celi Lopes Toledo, Elad Tako, Neuza Maria Brunoro Costa, Hércia Stampini Duarte Martino, Frederico Augusto Ribeiro de Barros

**Affiliations:** 1 Department of Food Technology, Federal University of Viçosa, Viçosa 36570-900, MG, Brazil; 2 Department of Nutrition and Health, Federal University of Viçosa, Viçosa 36570-900, MG, Brazil; 3 Department of Food Science, Cornell University, Stocking Hall, Ithaca, NY 14850, USA; 4 Department of Pharmacy and Nutrition, Federal University of Espírito Santo, Alegre 29500-000, ES, Brazil

**Keywords:** bioactive compounds, metabolic changes, oleic acid, carotenoid, tocopherol

## Abstract

Macauba is a palm tree native to Brazil, which fruits are rich in oil. Macauba pulp oil has high contents of oleic acid, carotenoids, and tocopherol, but its effect on health is unknown. We hypothesized that macauba pulp oil would prevent adipogenesis and inflammation in mice. Thus, the purpose of this study was to evaluate the effects of macauba pulp oil on the metabolic changes in C57Bl/6 mice fed a high-fat diet. Three experimental groups were used (*n* = 10): control diet (CD), high-fat diet (HFD), and high-fat diet with macauba pulp oil (HFM). The HFM reduced malondialdehyde and increased SOD activity and antioxidant capacity (TAC), showing high positive correlations between total tocopherol, oleic acid, and carotenoid intakes and SOD activity (r = 0.9642, r = 0.8770, and r = 0.8585, respectively). The animals fed the HFM had lower levels of PPAR-γ and NF-κB, which were negatively correlated with oleic acid intake (r = −0.7809 and r = −0.7831, respectively). Moreover, the consumption of macauba pulp oil reduced inflammatory infiltrate, adipocyte number and length, (mRNA) *TNF-α*, and (mRNA) *SREBP-1c* in the adipose tissue, and it increased (mRNA) *Adiponectin*. Therefore, macauba pulp oil prevents oxidative stress, inflammation, and adipogenesis and increases antioxidant capacity; these results highlight its potential against metabolic changes induced by an HFD.

## 1. Introduction

Current eating habits characterized by elevated consumption of saturated fats and simple carbohydrates and low vitamins are one of the most important causes for the emergence of chronic non-communicable diseases, such as obesity and nonalcoholic fatty liver disease [1,2]. Obesity is characterized by adipogenesis, which favors the induction of metabolic changes, including changes in cytokine concentrations, activation of inflammatory pathways, and lipotoxic effects in tissues such as the liver. As a consequence of these changes, reactive oxygen species (ROS) production may increase and may, thus, result in an exacerbation of inflammation, oxidative stress, and cell alterations [3].

Regulation and control of adipogenesis and metabolic changes are performed by specific transcriptional regulators, such as peroxisome proliferator-activated receptor gamma (PPAR-γ), sterol regulatory element-binding protein 1 (SREBP-1), and nuclear factor kappa B (NF-κB) [4]. SREBP-1 controls fatty acid biosynthesis by favoring the transcription of specific enzymes and activating PPAR-γ, which controls the expression of genes that regulate adipocyte differentiation. NF-κB controls the expression of inflammatory genes. In obesity, there is an increase in these transcription factors, resulting in increased lipogenesis, which leads to an increase in triacylglycerol and a reduction in lipolysis, thereby favoring the development of inflammation and oxidative stress [3,4]. As such, research studies that demonstrate new dietary strategies with the purpose of preventing or controlling obesity and metabolic alterations become very important, and dietary fatty acid composition demonstrates a significant impact on disease development [5]. Thus, nutritional strategies that aim to treat or prevent these metabolic alterations are of great importance.

Macauba (*Acrocomia aculeata*) is a palm tree that is naturally present in almost all Brazilian territories, and it is considered a promising alternative to vegetable oil for fuel and for the cosmetic and food industries due to its high oil production and specific characteristics [6]. Two types of oils are obtained from macauba: pulp and kernel oils. Both have important chemical and economical characteristics, highlighting their nutritional action and applications in the food industry [7]. Similar to olive oil, macauba pulp oil is rich in oleic acid [6]. Oleic acid has been shown to reduce the expression of transcription factors related to the adipogenesis signaling pathway, such as PPAR-γ, and reduce oxidative stress markers [8]. Moreover, macauba pulp oil has a high content of carotenoids, which can act to reduce inflammation through NF-κB modulation [6,9]. Additionally, this oil contains tocopherol, which is an important antioxidant that has been shown to improve inflammation and oxidative stress [10]. Thus, macauba pulp oil consumption may result in an improvement in metabolic changes, which is associated with the bioactive compounds in its composition [6].

We hypothesized that macauba pulp oil would prevent adipogenesis and inflammation in mice. However, to the best of our knowledge, no research has been performed to provide evidence of the health benefits of macauba pulp oil. Thus, the objective of this work was to evaluate the effect of macauba pulp oil on the adipogenesis pathways and metabolic changes in mice fed a high-fat diet. This study is the first to explore the health benefits of this promising vegetable oil.

## 2. Material and Methods

### 2.1. Materials

Macauba fruits were harvested in Araponga, Minas Gerais (Brazil), in the mature stage, and then they were peeled and pulped to obtain the macauba pulp. The pulp was dried at 65 °C (CIENLAB CE220, Brazil) for 15 h. Oil was extracted using a manual hydraulic press (Laboratory Press, Fred S. Carver Inc., Summit, NJ, USA), centrifuged (5000 rpm/20 min), and then placed in a freezer (−80 °C).

### 2.2. Chemical Characterization of Macauba Pulp Oil

The fatty acid profile of the macauba pulp oil was determined using a gas chromatography equipped with a flame ionization detector (GC-FID) (Shimadzu, GC-2010, Kyoto, Japan) and a capillary column of 100 m × 0.25 mm (SP-2560, Sigma-Aldrich, San Luis, MO, USA) [11]. Helium gas was used as the dragging gas and maintained at a constant flow rate of 363 kPa. Fatty acid methyl esters (FAMEs) were separated using a linear heating ramp from 100 °C to 270 °C, at a heating rate of 20 °C mim^−1^ and with a high linear velocity for better peak resolution. Peak identification was confirmed by comparison with the standard FAME mix (Supelco 37 FAME mix, Sigma-Aldrich, San Luis, MO, USA). Moreover, the oleic acid content (mg/g) of the oil was also determined using a standard (Sigma-Aldrich).

Carotenoid analysis was carried out by a high-performance liquid chromatography (HPLC) with detection at 450 nm, using the following chromatographic conditions: a HPLC system (Shimadzu, SCL 10AT VP, Kyoto, Japan) and a chromatographic column Phenomenex Gemini RP-18 (250 mm × 4.6 mm, 5 mm) fitted with a guard column RP-18 Phenomenex ODS column (4 mm × 3 mm). The mobile phase consisted of methanol:ethylacetate:acetonitrile (70:20:10, *v*/*v*/*v*) with a flow rate of 2.0 mL·min^−1^ and a run time of 15 min. Total carotenoid content (μg/g) was expressed as the sum of the major carotenoids present in the macauba pulp oil [12].

Total tocopherol content was determined following the AOCS method, using a HPLC with fluorescence detection at 450 nm and the following chromatographic conditions: a silica column of 4.6 × 250 mm with a pore of 5 μm, a flow rate of 1.0 mL min^−1^, and as the mobile phase, a mixture of 99.5% of *n*-hexane and 0.5% of isopropanol. The concentration of total tocopherols (μg/g) was expressed as the sum of the major tocopherols present in the macauba pulp oil [13].

### 2.3. Animals and Experimental Design

Black male mice C57Bl/6 (30 animals), which were 8 weeks old and had an average weight of 24.34 ± 0.18 g, were allocated into 3 groups, with 10 animals in each group, based on the homogeneity of body weight. The sample calculation equation determined how many animals should be in each group, using the following variables: α-error type I = 1.96, α-level = 5%, and data of fat mass mean reported by Schoemaker et al. in 2017 [14,15]. Individual stainless steel cages were used to keep the animals in a temperature-controlled room (light–dark cycles of 12 h and temperature of 22 ± 2 °C). Water and the respective experimental diets were supplied ad libitum.

The experimental diets were formulated according to AIN-93M and high-fat diet, using lard in the high-fat diet [16]. Each experimental group consumed the following diet: control diet—AIN93M (CD); high-fat diet (HFD); high-fat diet with macauba pulp oil (HFM). In the HFM, macauba pulp oil was added in a proportion of 40 g/kg (4%), replacing the soybean oil used in the AIN-93M diet (Table 1). The objective was to verify the effect of macauba pulp oil as a replacement of soybean oil, which is commonly used in control diets, and not as a supplementation. The formulated diets were stored at a low temperature (−20 °C) and offered to the animals every day.

At the end of the 8 weeks, the animals were anesthetized after 12 h of fasting using isoflurane (Isoforine, Cristália), in accordance with the bodyweight of the mice. Using the methodology of cardiac puncture, blood was collected and centrifuged (4 °C at 800× *g* for 10 min using Fanem-204, São Paulo, Brazil), and the serum was collected and stored at −80 °C. The liver and adipose (epididymal and subcutaneous) tissues were extracted and stored at (−80 °C) until analysis, and another part was fixed in formaldehyde (10%) for the analysis of histological markers. Bodyweight gain and feed consumption were measured on a weekly basis throughout the experiment to calculate the feed efficiency ratio (weight gain/consumption × 100), and the percentage of adiposity was measured based on the weight of the adipose tissue (g) in relation to the total body weight. Body mass index (BMI) was measured using the ratio between weight and naso-anal length (cm) squared [17]. The hepatosomatic index was also determined (liver weight/body weight × 100) [18]. Carotenoid, oleic acid, and tocopherol intakes were determined by the total amount of diet consumed by the mice. Ethical principles for animal experimentation were implemented for all processes performed on the animals [19]. The Ethics Committee of the Federal University of Viçosa approved this research (Protocol 09/2019; date of approval: 28 May 2019).

### 2.4. Biochemical Analysis

The biochemical parameters were determined using the serum. Glucose concentration, total cholesterol (TC), high-density lipoprotein cholesterol (HDL-c), low-density lipoprotein cholesterol (LDL-c), triacylglycerides (TGL), aspartate aminotransferase (AST), and alanine aminotransferase (ALT) were determined based on the colorimetric method using commercial kits (Bioclin^®^, Belo Horizonte, Brazil).

### 2.5. Homogenate Preparation and Oxidative Stress Levels

Liver homogenate was prepared with 200 mg of the liver. The liver was mixed with 1 mM of EDTA (pH 7.4) and 1000 μL of phosphate buffer (50 mM). The content was macerated and centrifuged (1200× *g*/8 min/4 °C), and the supernatant was collected for the analysis of antioxidant enzymes.

For the quantification of the enzyme superoxide dismutase (SOD), 249 μL of 50 mM of Tris-HCl buffer (pH 8.2) (1 mM of EDTA, 6 μL of MTT (1.25 mM), 15 μL of pyrogallol (10 mM), and 279 μL of buffer) was mixed into the aliquoted homogenate. To determine the blank, 6 μL of MTT and 294 μL of buffer were added to the wells, which were incubated for 5 min at 37 °C, and the reading was performed on a spectrophotometer at 570 nm (Thermo Scientific Multiskan GO, Waltham, MA, USA). The SOD quantification was expressed as units of SOD/mg protein [20].

Malondialdehyde (MDA) was determined using the samples of the homogenate. A total of 400 μL of trichloroacetic acid solution (15%) and thiobarbituric acid (0.375%) was added into 400 μL of the sample. It was placed in a water bath (90 °C/40 min) and 600 μL of n-Butanol was added; then, the mixture was centrifuged (3500 rpm/ 5 min). The supernatant was removed, and the absorbance was read at 535 nm (Multiskan GO—Thermo Scientific). The MDA level was expressed as MDA/mg protein [21].

Catalase was performed on the samples of the homogenate as described above. At 0, 30, and 60 s after the reaction was initiated, the absorbance was determined at 240 nm (T70 + UV/VIS Spectrometer). Enzyme activity was reported as μmol per mL of sample, and the data were expressed in U of catalase/mg protein. Catalase activity was calculated according to the Beer–Lambert law [22].

For the quantification of nitric oxide, 50 μL of the homogenate was used. Then, 1% sulfanilamide solution and 0.1% nafityl ethylene amide dihydrochloride were added. A 0.025 M sodium nitrite standard curve was used, and the absorbance was determined at 570 nm (Multiskan GO—Thermo Scientific) [23].

### 2.6. Total Antioxidant Capacity of Serum and Liver

The total antioxidant capacity (TAC) of the serum and the liver was determined with an antioxidant assay kit (Cayman Chem Corp, Ann Arbor, MI, USA) Sigma Aldrich^®^. The absorbance reading was performed at 405 nm (Multiskan GO—Thermo Scientific).

### 2.7. PPAR-γ, PPAR-α, NF-κB, and TLR-4 Quantification

The adipose tissue and liver samples were homogenized using the NE-PER™ Nuclear and Cytoplasmic Extraction Kit reagents (Thermo Scientific Fisher, Waltham, MA, USA). The nuclear fractions were analyzed using an immunoassay with the Mouse PPAR-γ (Peroxisome Proliferator-Activated Receptor Gamma—E-EL-M0893, Elabscience, Houston, TX, USA), Mouse NF-κB p65 (Factor Nuclear Kappa B—E-EL-M0838, Elabscience, Houston, TX, USA), Rat PPAR-α (Peroxisome Proliferator-Activated Receptor Alfa—E-EL-R0725, Elabscience, Houston, TX, USA), Rat NF-κB p65 (Factor Nuclear Kappa B—E-EL-R0674, Elabscience, USA) and Rat TLR-4 (Toll-like Receptor 4—E-EL-R0990, Elabscience, USA) ELISA kits, respectively. The microplates were, respectively, precoated with anti-PPAR-γ, anti-NF-κB p65, anti-PPAR-α, and anti-TLR-4 antibodies. The concentrations were calculated by comparison to the corresponding standard curves.

### 2.8. Determination of Gene Expression in Adipose Tissue and Liver by Reverse Transcriptase Quantitative Polymerase Chain Reaction (RT-qPCR)

TRIzol reagent (Invitrogen, CA, USA) was used to extract total RNA from the liver, and a specific kit (mirVana™ miRNA Isolation Kit, Life Technologies, Carlsbad, CA, USA) was used to extract RNA from the adipose tissue, according to the manufacturer’s protocols. RNA concentration and purity were evaluated using a Microdrop plate spectrophotometer Multiskan™ GO (Thermo Scientific, Waltham, MA, USA). To create cDNA synthesis, the M-MLV Reverse Transcriptase Kit (Invitrogen, CA, USA) was used. RT-qPCR was used for the gene expression relative quantification using the AB StepOne Real-Time PCR System equipment and Fast SYBR Green Master Mix (Applied Biosystems, Carlsbad, CA, USA) reagent. The initial parameters used were 20 s at 95 °C and then 40 cycles at 95 °C (3 s), 60 °C (30 s), followed by the melting curve analysis. A melting point analysis was performed to improve the specificity and sensitivity of the amplification reactions detected. All primers were designed by using the Primer 3 Plus program and obtained from Sigma-Aldrich Brazil Ltda. (Table 2). The 2-Delta-Delta C (T) method was used to calculate the gene expression, by using GAPDH and β-actin as the references and the high-fat diet group as the control, which was normalized to 1 [24].

### 2.9. Histomorphometric Analysis of Adipose and Liver Tissues

Paraffin was used to fix the samples of adipose tissue and liver. Ten cuts per animal were performed (3 μm thick), and the samples were mounted on glass slides and stained with hematoxylin and eosin. Analyses were performed under a light microscope (Leica DM750^®^). The histological sections of the images were captured in a 20× objective. Inflammatory infiltrate number and length of adipocytes were evaluated using the adipose tissue (Image-Pro Plus^®^ 4.5). Liver cellular components (fat vesicles, inflammatory infiltrate, cytoplasm, and nucleus), for 10 histological fields per animal, were analyzed using a test system with 266 points, obtaining 2660 total points for each animal analyzed (Image J^®^, Wayne Rasband). The following formula was used to calculate the parameters: *V*_v_ = *P*_p_/*P*_T_ (*P*_p_ = number of points located on the structure of interest, and *P*_T_ = total test points in the histological area) [25]. The steatosis degree was determined semi-quantitatively according to a 5° scale and the fat percentage: degree 0 (<5%), grade 1 (≥5% and <25%), grade 2 (≥25% and <50%), grade 3 (≥50% and <75%), grade 4 (≥75%) [26].

### 2.10. Statistical Analyses

Kolmogorov–Smirnov normality test was initially applied, and then an analysis of variance (ANOVA) test was performed, followed by the Newman–Keuls test for parametric variables. For the correlation analysis, Pearson’s correlation was used. The results with a *p*-value ≤ 0.05 were considered statistically significant. The statistical analyses were performed using the GraphPad Prism^®^ version 8.0 (GraphPad Software, San Diego, CA, USA).

## 3. Results

### 3.1. Chemical Characterization of Macauba Pulp Oil

The macauba pulp oil shows a high content of monounsaturated fatty acids (55%), with significant oleic acid content (49.32%), as shown in Table 3. In addition, it has high contents of carotenoids and tocopherol (Table 3).

### 3.2. Effects of Macauba Pulp Oil on Biometric Measures, Food Intake, and Lipid Profile

Weight gain, body mass index (BMI), and food efficiency ratio (FER) did not differ among the experimental groups (*p* > 0.05; Table 4). The CD group had higher food consumption compared to the HFD and HFM groups, which was associated with the reduced caloric density of the AIN93M diet (*p* < 0.0001; Table 4). The CD group had a lower percentage of adiposity compared to the HFD and HFM groups (*p* = 0.0018; Table 4).

The group that consumed macauba pulp oil (HFM) did not differ from the HFD group in terms of glucose, triglyceride, TC, LDL, and HDL values, as well as hepatic enzymes AST and ALT, and hepatosomatic index (*p* > 0.05; Table 4).

### 3.3. Total Antioxidant Capacity and Oxidative Stress Marker Levels in Mice

**Figure 1:**
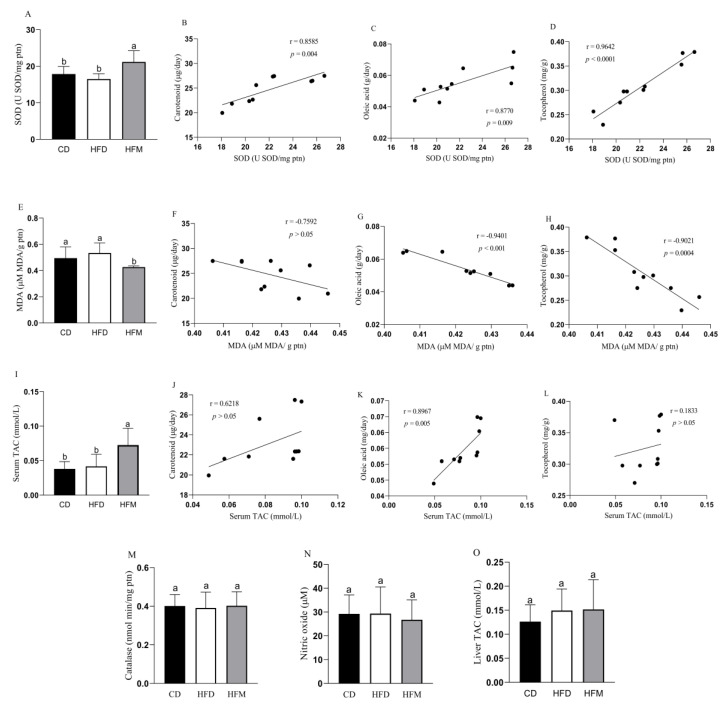
Oxidative stress level and antioxidant capacity of mice after consuming the experimental diets for 8 weeks, and correlation with carotenoid, oleic acid, and tocopherol intakes. (**A**) Superoxide dismutase level. (**B**) Correlation between SOD level and carotenoid intake. (**C**) Correlation between SOD level and oleic acid intake. (**D**) Correlation between SOD level and tocopherol intake. (**E**) Malondialdehyde level. (**F**) Correlation between MDA level and carotenoid intake. (**G**) Correlation between MDA level and oleic acid intake. (**H**) Correlation between MDA level and tocopherol intake. (**I**) Serum total antioxidant capacity level. (**J**) Correlation between serum TAC level and carotenoid intake. (**K**) Correlation between serum TAC level and oleic acid intake. (**L**) Correlation between serum TAC level and tocopherol intake. (**M**) Catalase level. (**N**) Nitric oxide level. (**O**) Liver total antioxidant capacity level. Data are expressed as mean ± standard deviation (*n* = 10). Different letters indicate a statistical difference based on the Newman–Keuls test (*p* ≤ 0.05). CD: control diet—AIN93M; HFD: high-fat diet; HFM: high-fat diet with macauba pulp oil; SOD: superoxide dismutase; MDA: malondialdehyde; TAC: total antioxidant capacity.

The HFM group had a high SOD activity (*p* = 0.0078; Figure 1A) and showed a positive correlation with carotenoid (r = 0.8585, *p* = 0.004), oleic acid (r = 0.8770, *p* = 0.009) and tocopherol (r = 0.9642, *p* < 0.0001) intakes (Figure 1B–D). Macauba pulp oil decreased malondialdehyde (*p* = 0.0057; Figure 1E), showing a negative correlation between this parameter and oleic acid (r = −0.9401, *p* < 0.001) and tocopherol (r = −0.9021, *p* = 0.0004) (Figure 1G,H). Catalase and nitric oxide did not differ among the groups (*p* > 0.05; Figure 1M,N).

The HFM group had a higher serum TAC compared to the HFD and CD groups (*p* = 0.0058; Figure 1I), showing a positive correlation between serum TAC and oleic acid (r = 0.8967, *p* = 0.005) intake from macauba pulp oil (Figure 1K). Liver TAC did not differ among the experimental groups (*p* > 0.05; Figure 1O).

### 3.4. Effects of Macauba Pulp Oil on NF-κB, TLR-4, and PPAR-(α, γ) Quantification

**Figure 2:**
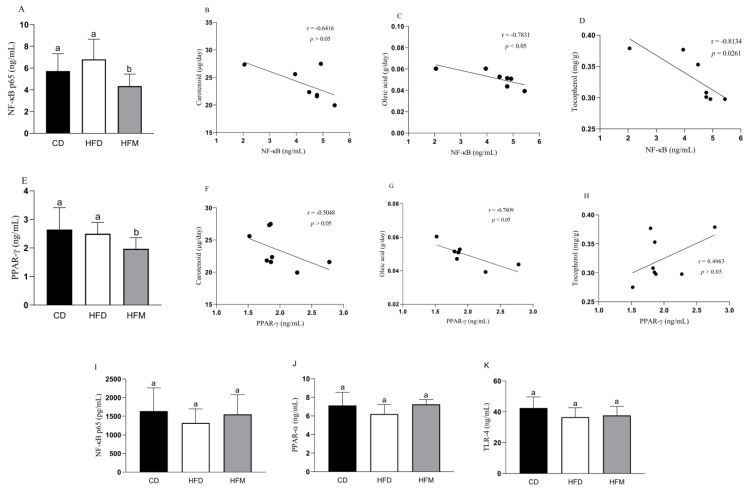
Levels of proteins in the adipose tissue (**A**–**H**) and liver (**I**–**K**) of mice after consuming the experimental diets for 8 weeks, and correlation with carotenoid, oleic acid, and tocopherol intakes. Data are expressed as mean ± standard deviation (*n* = 8). Different letters indicate a statistical difference based on the Newman–Keuls test (*p* ≤ 0.05). CD: control diet—AIN93M; HFD: high-fat diet; HFM: high-fat diet with macauba pulp oil; NF-κB p65: nuclear factor kappa B subunit p65; PPAR-α: peroxisome proliferator-activated receptor alpha; PPAR-γ: peroxisome proliferator-activated receptor gamma; TLR-4: toll-like receptor 4.

The HFM group had a lower nuclear quantification of NF-κB in the adipose tissue compared to the HFD and CD groups (*p* = 0.0179; Figure 2A), showing a negative correlation with oleic acid (r = −0.7831, *p* = 0.037) and tocopherol (r = −0.8134, *p* = 0.0261) intakes (Figure 2C,D). Macauba pulp oil reduced the PPAR-γ quantification (*p* = 0.056; Figure 2E), showing a negative correlation with carotenoid (r = −0.7301, *p* = 0.021) and oleic acid (r = −0.7809, *p* = 0.022) (Figure 2F,G).

NF-κB, PPAR-α, and TLR-4, as present in the nuclear fraction in the liver, did not differ among the experimental groups (*p* > 0.05; Figure 2I–K).

### 3.5. Effects of Macauba Pulp Oil on Gene Expression in Adipose and Hepatic Tissues

**Figure 3:**
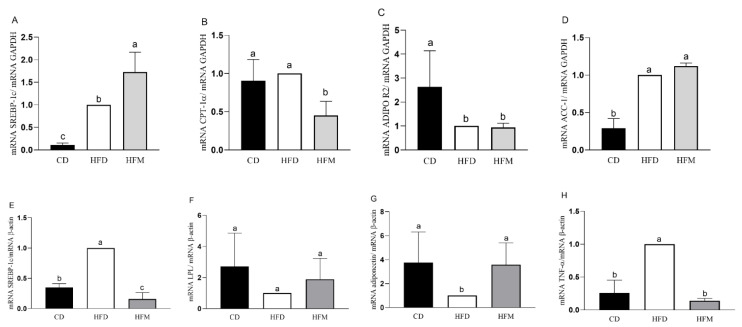
Gene expression in the liver (**A**–**D**) and adipose tissue (**E**–**H**) of mice after consuming the experimental diets for 8 weeks. Data are expressed as mean ± standard deviation (*n* = 8). Different letters indicate a statistical difference based on the Newman–Keuls test (*p* ≤ 0.05). CD: control diet—AIN93M; HFD: high-fat diet; HFM: high-fat diet with macauba pulp oil; *SREBP-1c*: sterol regulatory element-binding proteins 1c; *ADIPOR2*: adiponectin receptor 2; *ACC-1*: acetyl CoA carboxilase 1; *CPT-1α*: carnitine palmitoyl transferase 1 alpha; *GAPDH*: glyceraldehyde-3-phosphate dehydrogenase; *LPL*: lipoprotein lipase; *TNF-α*: tumor necrosis factor alpha.

In the liver, in the HFM group, the mRNA expression of *SREBP-1c* was significantly increased compared to the control and HFD groups (*p* < 0.0001; Figure 3A), whereas (mRNA) *CPT-1α* was decreased (*p* = 0.0031; Figure 3B). The mRNA expression of *ACC-1α* and *AdipoR2* did not differ from the HFD group (*p* > 0.05; Figure 3C,D).

In the adipose tissue, in the HFM group, the mRNA expression of *SREBP-1c* (*p* < 0.0001; Figure 3E) and (mRNA) *TNF-α* (*p* < 0.0001; Figure 3H) were significantly decreased compared to the HFD group, and the mRNA expression of *Adiponectin* was similar between the HFM and CD groups (*p* > 0.05; Figure 3G). The mRNA expression of *LPL* was similar among the groups (*p* > 0.05; Figure 3F). The correlation analysis showed a negative correlation between mRNA *SREBP-1c* and carotenoid intake (r = −0.8991, *p* = 0.012), a positive correlation between mRNA *Adiponectin* and carotenoid intake (r = 0.9130, *p* < 0.001), and negative correlation between mRNA *TNF-α* and oleic acid intake (r = −0.9057, *p* = 0.0009).

### 3.6. Effects of Macauba Pulp Oil on Histological Morphometrics of Liver and Adipose Tissues

**Figure 4:**
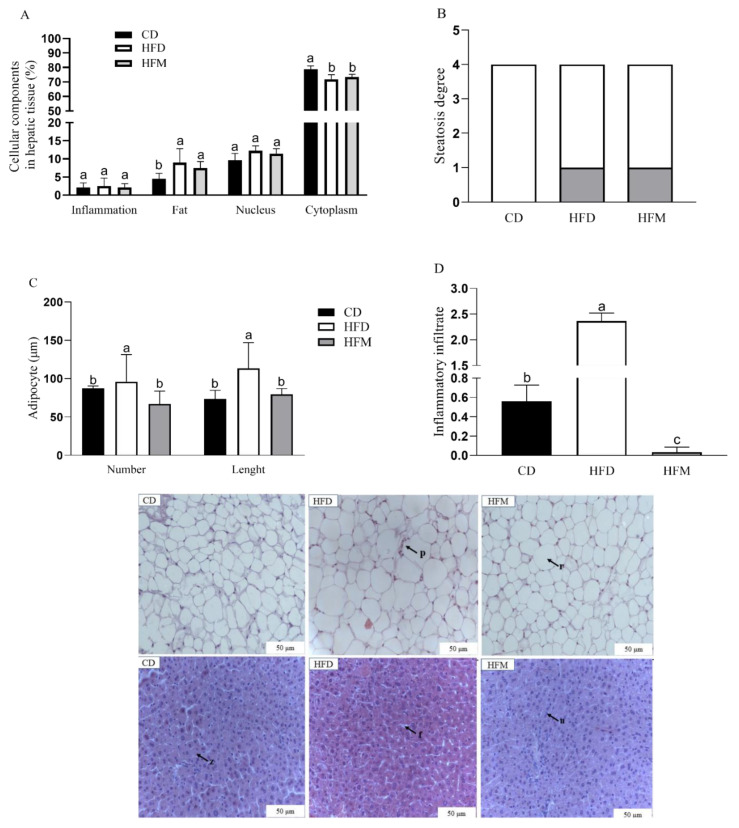
Cellular components: percentage in hepatic tissue (**A**), steatosis degree (**B**), number and length of adipocyte (**C**), and inflammatory infiltrate (**D**). The black arrows represent the following: z: cytoplasm, f: fat vesicles, n: nucleus, p: inflammatory infiltrate, and r: adiposity. Data are expressed as mean ± standard deviation (*n* = 10). Different letters indicate a statistical difference based on the Newman–Keuls test (*p* ≤ 0.05). CD: control diet; HFD: high-fat diet; HFM: high-fat diet with macauba pulp oil.

The percentage of the nucleus, cytoplasm, inflammatory infiltrate, and fat deposition in the hepatocytes did not differ among the groups (*p* > 0.05, Figure 4A). The control group was identified as steatosis grade 0, whereas the HFD and HFM groups increased the steatosis to grade 1 and had similar values between them (Figure 4B). The HFM group had lower inflammatory infiltrate (*p* < 0.0001) and adipocyte number (*p* = 0.0027) and length (*p* = 0.0088) in the adipose tissue compared to the HFD group, but its values were similar to the CD group (Figure 4C,D).

## 4. Discussion

This is the first work that evaluated the influence of macauba pulp oil on undesirable metabolic changes in mice fed a high-fat diet. The present research focused on the effects of macauba pulp oil since there is evidence that oleic acid, carotenoid, and tocopherol present in this oil would trigger anti-inflammatory, anti-obesity, and antioxidant effects [27,28]. In this study, macauba pulp oil intake prevented the adipogenesis pathway, inflammation, and oxidative stress in mice fed a high-fat diet. In order to stimulate metabolic changes in animals, high-saturated fat diet consumption is extensively applied. The time to verify the effect of a specific food or compound on metabolic changes usually begins after seven or eight weeks of receiving the diet. In a different way, in our study, to determine the effects of macauba pulp oil as a preventive treatment, macauba pulp oil was added in the diet since the beginning of the experiment, along with the high-fat diet, to examine its mechanism of action and metabolic alterations.

In the present study, the consumption of macauba pulp oil reflected a higher total antioxidant capacity (TAC), which might be associated with the oleic acid content, and this was confirmed by the correlation analysis, which demonstrated a significant positive correlation between this compound consumption and TAC. Oleic acid is well documented for its anti-inflammatory properties, possibly associated with its chemical configuration with a double bond, thereby causing less chance of oxidation and resulting in the antioxidant property against a high oxidative load [10,29]. In addition, higher SOD activity and lower malondialdehyde levels were observed with the macauba pulp oil consumption. SODs are oxidoreductase enzymes that have a role in protecting cells against superoxide anions, performing the dismutation of O_2_^•−^ into oxygen and H_2_O_2_, and providing antioxidant defense for the organism, while malondialdehyde is an important marker of lipid peroxidation [30,31]. it is shown that macauba pulp oil consumption can improve antioxidant defenses, with these results being attributed to the oleic acid, carotenoids, and tocopherol present in macauba pulp oil, as demonstrated in other studies that examined the relationship between these components and the improvement of the body’s antioxidant defenses [32,33,34]. Additionally, there was a positive correlation between SOD and these compounds and a negative correlation between MDA and oleic acid and tocopherol.

The consumption of macauba pulp oil prevented the adipogenesis pathway by decreasing the expression of PPAR-γ and (mRNA) *SREBP-1c* and increasing the expression of (mRNA) *Adiponectin* in the adipose tissue. This effect was confirmed by the result of the histomorphometric analysis, which demonstrated that the animals that consumed the macauba pulp oil had smaller adipocyte number and length even with a high-fat diet consumption, that is, the macauba pulp oil caused less hypertrophy and hyperplasia of the adipocytes. Thus, the lower translocation of PPAR-γ in the present research could be associated with the high content of oleic acid and carotenoids in the macauba pulp oil and was confirmed by the significant negative correlation between the consumption of these compounds and PPAR-γ quantification. Oleic acid has been shown to act in PPAR-γ repression, resulting in less differentiation of pre-adipocytes into mature adipocytes and reducing adipogenesis [3,35]. Similar to our results, a previous study found a relationship between oleic acid consumption and reduction in PPAR-γ and (mRNA) *SREBP-1c* in an obese animal model [35]. Research shows that carotenoids can affect adipocyte function through the interaction with PPAR-γ, thereby interfering with adipocyte differentiation, as demonstrated in a study using experimental animals, which found an association between carotenoids and lower adipose tissue gain related to lower PPAR-γ expression [36]. Still, this result is related to the increased expression of adiponectin since PPAR-γ is tightly regulated by adiponectin [37]. Moreover, the observed results of a reduction in the genes related to the adipogenesis pathway, with a concomitant reduction in the histological markers of adipose tissue, could be related to the presence of β-carotene, which was the main carotenoid found in the macauba pulp oil that could suppress PPAR-γ, resulting in lower total lipid in adipocytes [38,39].

Related to this, macauba pulp oil was efficient in reducing inflammation in the adipose tissue since it reduced NF-κB in the nuclear fraction, and this indicates a reduction in the inflammation cascade, leading to a significant reduction in (mRNA) *TNF-α* gene expression. Corroborating this result, the histomorphometric analysis of the adipose tissue showed less inflammatory infiltrate with the consumption of macauba pulp oil. A hypertrophy of adipose tissue initiates the emission of chemotactic signals that recruit immune cells and lead to the infiltration of macrophages into the adipose tissue, contributing to systemic subclinical inflammation [3]. This result may be associated with a lower amount of PPAR-γ and higher adiponectin since PPAR-γ interferes with the differentiation of adipocytes and is consequently related to the inflammatory process. Obesity is an inflammatory condition: one of the complications related to obesity is the development of reactive oxygen species (ROS), and adiponectin is an anti-inflammatory adipokine with a negative correlation between the degree of obesity and the level of this adipokine [40,41]. These results were supported by the present study since there was a positive correlation between carotenoid consumption and an increase in the expression of adiponectin, indicating that the macauba pulp oil, which is high in carotenoids, may contribute to the reduction of inflammation. Additionally, there was a significant negative correlation between oleic acid and tocopherol and NF-κB, that is, an increase in oleic acid and tocopherol consumption was correlated with a decrease in the quantification of NF-κB. The study by Rosillo et al., with a mouse model, also demonstrated that the administration of oleic acid is able to suppress NF-κB activation [42]. Oleic acid is able to activate PGC-1α by forming a dimer with the protein called c-MAF, migrate to the nucleus, and then transcribe the gene responsible for IL-10, which dismantles the activation signaling of NF-κB due to its potent anti-inflammatory action [43]. Tocopherol can block NF-kB activation through its action on enzymes that regulate the NF-kB signaling pathway [44]. Despite the lack of a correlation between the reduction in NF-κB and the consumption of carotenoids in the present study, this compound presents interference with the NF-κB pathway, resulting in the modulation of their interacting proteins and interacting with the cysteine residues of IκB kinase, thereby suppressing NF-κB activation or inhibiting IκBα degradation [45,46].

Although macauba pulp oil prevented the adipogenesis pathway and inflammation in the adipose tissue, significant effects in the hepatic markers were not observed after eight weeks of the high-fat diet. The current study was carried out as a prevention model, and for this reason, it might not be able to verify alterations in the liver. Thus, in the current research, the consumption of the diets for eight weeks, even with a high concentration of saturated fats, was not able to cause metabolic changes in the liver. These results were confirmed by histomorphometric analyses, which showed that there was no alteration of the cellular components evaluated, such as fat and inflammation in the liver. 

Despite the decreased expression of (mRNA) *CPT-1α* gene, the quantification of PPAR-α did not change with the consumption of macauba pulp oil, which might be because ADIPOR2 did not change either. The increase in the sensitization of ADIPOR2 receptor triggers the activation of PPAR-α, which regulates fatty acid oxidation [47]. Moreover, the high traffic of free fatty acids due to a high-fat diet has the ability to trigger SREBP-1c, which controls the expression of enzymes essential in triacylglycerol synthesis and storage, and restricts lipogenic genes, such as ACC-1, that are responsible for the transformation of ACC-1 to malonyl CoA [48]. However, despite the overexpression of this gene in the fatty acid synthesis pathway, there was no change in the proportion of fat and steatosis degree in the liver. This might be due to the increased antioxidant capacity, which decreased the expression of this gene in relation to fatty acid synthesis. 

The strain of mice used in this study was chosen since they are prone to metabolic disturbances generated by a high-fat diet. However, it is known that experiments with mice do not fully reflect the effects in humans due to differences in the organs and metabolism of these two species. However, taking into account the macauba pulp oil intake per animal weight, a human with 70 kg needs a consumption of a small amount per day (approximately 8 g/day of macauba pulp oil—similar to one teaspoon) to have the same improvements observed in this research in the prevention of metabolic changes. Thus, further studies are needed to verify the real effects of macauba pulp oil in human.

**Figure 5:**
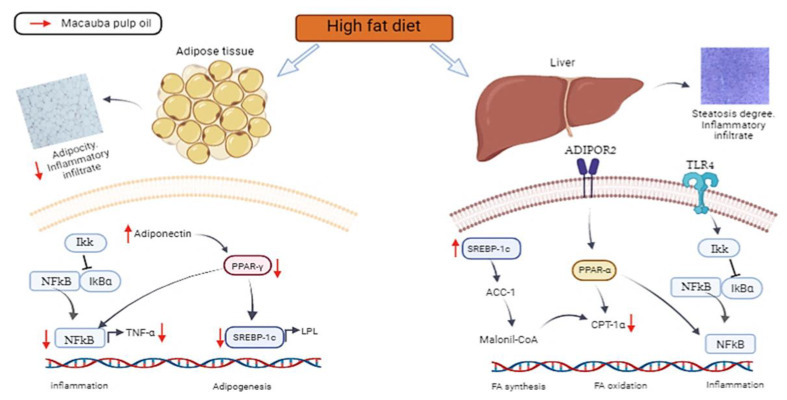
Effects of a high-fat diet on the adipose tissue and liver and potential action mechanism of macauba pulp oil. TLR-4: toll-like receptor 4; NF-κB: nuclear factor kappa B; *SREBP-1c*: sterol regulatory element-binding protein; *ACC-1*: acetyl-CoA carboxylase 1; *ADIPOR2*: adiponectin receptor 2; *CPT-1α*: carnitine palmitoyl transferase 1 alpha; *PPAR-γ*: peroxisome proliferator-activated receptor gamma; PPAR-α: peroxisome proliferator-activated receptor alpha; *LPL*: lipoprotein lipase; *TNF-α*: tumor necrosis factor alpha; Ikk: IkB kinase complex; IkBα nuclear factor of kappa light polypeptide gene enhancer in B-cells inhibitor alpha; Malonil-CoA: malonyl coenzyme A; FA: fatty acid.

The influence of a high-fat diet on the body and the mechanism of macauba pulp oil, which was demonstrated in our study, are summarized in Figure 5. The consumption of macauba pulp oil prevents inflammation and adipogenesis, as demonstrated by a reduction in the expression of PPAR-γ, (mRNA) *SREBP-1c*, NF-κB, and (mRNA) *TNF-α*, and an increase in adiponectin in adipose tissue. In the liver, despite triggering the *SREBP-1c* expression and a lower (mRNA) *CPT-1α* level, it does not lead to liver changes, according to the histomorphometric analysis, due to an increased antioxidant capacity. These modes of action may be related to macauba pulp oil, which has a good composition of carotenoids, oleic acid, and tocopherol and improves the total antioxidant capacity, resulting in adipogenesis even with a high level of saturated fat consumption.

## 5. Conclusions

Consumption of macauba pulp oil increases antioxidant capacity and prevents oxidative stress, inflammation, and the adipogenesis pathway. Therefore, macauba pulp oil has a great potential for inclusion in human foods to improve health, assisting in the prevention of risk factors for chronic non-communicable diseases.

## Figures and Tables

**Table 1:** Composition of experimental diets (g/kg of diet).

Ingredients (g/kg)	CD	HFD	HFM
Albumin *	179.71	179.71	179.71
Dextrinized starch	155	155	155
Sucrose	100	100	100
Soybean oil	40	40	-
Lard	0	312	312
Cellulose	50	50	50
Mineral mix	35	35	35
Vitamin mix	10	10	10
L-cystine	1.8	1.8	1.8
Choline bitartrate	2.5	2.5	2.5
Corn starch	425.99	113.99	113.99
Macauba pulp oil	-	-	40
Carbohydrate (%)	76.9	44.1	44.1
Protein (%)	18.9	18.9	18.9
Lipids (%)	4.20	37	37
Caloric density (kcal g^−1^)	3.85	5.41	5.41

* Purity of 78%. CD: control diet (AIN93M); HFD: high-fat diet; HFM: high-fat diet with macauba pulp oil.

**Table 2:** Sequence of primers used in the RT-qPCR analyses.

Genes	Forward	Reverse
*SREBP-1c*	CGC TAC CGT TCC TCT ATC AAT GAC	AGT TTC TGG TTG CTG TGC TGT AAG
*ADIPOR2*	CAT GTT TGC CAC CCC TCA GTA	ATG CAA GGT AGG GAT TCC A
*ACC-1*	TCA AGA CGG CTC AGG TCA TCA	AGG CGC CAA ACT TCA GCA TC
*CPT-1α*	GTA AGG CCA CTG ATG AAG GAA GA	ATT TGG GTC CGA GGT TGA CA
*LPL*	TCA ACC ACA GCA GCA AGA	CCG ATA CAA CCA GTC TAC TAC AA
*Adiponectin*	ATG AGT ACC AGA CTA ATG AGA C	GGC AGG ATT AAG AGG AAC A
*TNF-α*	TAT GGC TCA GGG TCC AAC TC	GCT CCA GTG AAT TCG GAA AG
*SREBP-1c*	GCC GAG ATG TGC GAA CTG	GGA AGT CAC TGT CTT GGT TGT T
*β-actin*	TTC GTT GCC GGT CCA CC	GCT TTG CAC ATG CCG GAG CC
*GAPDH*	AGG TTG TCT CCT GTC ACT TC	CTG TTG CTG TAG CCA TAT TC

*SREBP-1c*: Sterol regulatory element-binding proteins 1c; *ADIPOR2*: adiponectin receptor 2; *ACC-1*: acetyl CoA carboxylase 1; *CPT-1α*: carnitine palmitoyl transferase 1 alpha; *LPL*: Lipoprotein lipase; *TNF-α*: Tumor necrosis factor alpha; *GAPDH*: Glyceraldehyde-3-phosphate dehydrogenase.

**Table 3:** Fatty acid profile, carotenoids, and tocopherol contents in macauba pulp oil.

Components	
Palmitic (C16:0)	22.84%
Palmitoleic (C16:1)	5.93%
Stearic (C18:0)	1.23%
Oleic (C18:1n9c)	49.32%
Linoleic (C18:2n6c)	19.63%
Linolenic (C18:3n6c)	1.05%
Oleic acid (mg/g)	199.00
Tocopherol (μg/g)	40.80
Total carotenoids (μg/g)	207.52
β-carotene (μg/g)	163.63
α-carotene (μg/g)	21.03
Lutein (μg/g)	8.75
Lycopene (μg/g)	14.11

Caprylic, capric, lauric, and myristic acids are not detected.

**Table 4:** Biometric measures, food intake, and serum biochemical values of the mice after consuming the experimental diets for 8 weeks.

	CD	HFD	HFM
Weight gain (g)	4.01 ± 1.74 ^a^	4.14 ± 2.23 ^a^	3.66 ± 2.23 ^a^
BMI (g/cm^2^)	0.34 ± 0.02 ^a^	0.33 ± 0.01 ^a^	0.33 ± 0.02 ^a^
Adiposity (%)	0.71 ± 0.24 ^b^	2.43 ± 1.28 ^a^	2.26 ± 1.25 ^a^
Food consumption (g/day)	4.07 ± 0.16 ^a^	2.53 ± 0.41 ^b^	2.63 ± 0.42 ^b^
Food efficiency (%)	1.69 ± 0.60 ^b^	2.67 ± 1.46 ^a^	2.42 ± 1.55 ^a^
Hepatosomatic index (%)	3.61 ± 0.29 ^a^	3.72 ± 0.28 ^a^	3.48 ± 0.29 ^a^
Oleic acid intake (mg/day)	-	-	0.52 ± 0.08
Carotenoid intake (µg/day)	-	-	21.96 ± 4.11
Tocopherol intake (mg/day)	0.46 ± 0.01 ^a^	0.29 ± 0.03 ^b^	0.31 ± 0.03 ^b^
Total cholesterol (mg dL^−1^)	151.48 ± 13.79 ^b^	166.49 ± 15.51 ^a^	179.91 ± 6.87 ^a^
Total triglycerides (mg dL^−1^)	79.91 ± 4.71 ^a^	84.83 ± 5.63 ^a^	83.06 ± 5.09 ^a^
HDL-c (mg dL^−1^)	38.13 ± 4.29 ^a^	37.35 ± 5.79 ^a^	43.07 ± 5.64 ^a^
LDL-c (mg dL^−1^)	12.80 ± 2.11 ^b^	20.64 ± 5.25 ^a^	20.80 ± 5.20 ^a^
Glucose (mg dL^−1^)	160.67 ± 44.23 ^a^	182.58 ± 30.09 ^a^	197.31 ± 36.68 ^a^
AST (mg dL^−1^)	88.14 ± 21.88 ^a^	71.66 ± 21.30 ^a^	73.39 ± 19.04 ^a^
ALT (mg dL^−1^)	18.74 ± 9.88 ^a^	15.71 ± 5.63 ^a^	18.84 ± 9.26 ^a^

Data are expressed as mean ± standard deviation (*n* = 10). Different lowercase letters in the same row indicate a statistical difference based on the Newman–Keuls test (*p* ≤ 0.05). CD: control diet—AIN93M; HFD: high-fat diet; HFM: high-fat diet with macauba pulp oil; BMI: body mass index; HDL-c: high-density lipoprotein; LDL-c: low-density lipoprotein; ALT: alanine aminotransferase; AST: aspartate aminotransferase.

## Data Availability

Data are contained within the article.
